# Wicked problems in a post-truth political economy: a dilemma for knowledge translation

**DOI:** 10.1057/s41599-023-01789-6

**Published:** 2023-06-03

**Authors:** Matthew Tieu, Michael Lawless, Sarah C. Hunter, Maria Alejandra Pinero de Plaza, Francis Darko, Alexandra Mudd, Lalit Yadav, Alison Kitson

**Affiliations:** 1 College of Nursing and Health Sciences, Flinders University, Bedford Park, SA Australia; 2 Caring Futures Institute, Flinders University, Bedford Park, SA Australia; 3 College of Humanities, Arts and Social Sciences, Flinders University, Bedford Park, SA Australia; 4 College of Health, Wellbeing and Life Sciences, Sheffield Hallam University, Sheffield, UK

**Keywords:** Science, technology and society, Philosophy, Complex networks

## Abstract

The discipline of knowledge translation (KT) emerged as a way of systematically understanding and addressing the challenges of applying health and medical research in practice. In light of ongoing and emerging critique of KT from the medical humanities and social sciences disciplines, KT researchers have become increasingly aware of the complexity of the translational process, particularly the significance of culture, tradition and values in how scientific evidence is understood and received, and thus increasingly receptive to pluralistic notions of knowledge. Hence, there is now an emerging view of KT as a highly complex, dynamic, and integrated sociological phenomenon, which neither assumes nor creates knowledge hierarchies and neither prescribes nor privileges scientific evidence. Such a view, however, does not guarantee that scientific evidence will be applied in practice and thus poses a significant dilemma for KT regarding its status as a scientific and practice-oriented discipline, particularly within the current sociopolitical climate. Therefore, in response to the ongoing and emerging critique of KT, we argue that KT must provide scope for relevant scientific evidence to occupy an appropriate position of epistemic primacy in public discourse. Such a view is not intended to uphold the privileged status of science nor affirm the “scientific logos” per se. It is proffered as a counterbalance to powerful social, cultural, political and market forces that are able to challenge scientific evidence and promote disinformation to the detriment of democratic outcomes and the public good.

## Introduction

### The origin of knowledge translation

As health and medical research continues to generate evidence (or knowledge) that may give rise to new technologies or ways to address current and emerging healthcare challenges, the case is made that such evidence needs to be translated into clinical and healthcare practice^1^. Recent history indicates that this has not been an easy task with only around 50% of people receiving care in line with evidence-based guidelines (Braithwaite et al., 2020; McGlynn et al., 2003; Runciman et al., 2012). Where evidence has been translated into practice, it is often slow and haphazard, and in the context of public policy and legislation, the process can often be more complex and take much longer (Balas and Boren, 2000; Morris et al., 2011; Rushmer et al., 2019). Thus, the impetus for the emergence of the field of knowledge translation (KT) and its continual evolution and refinement arose. KT emerged from two distinct ways of understanding how scientific evidence or knowledge could be applied in practice, a positivist empiricist view, and a sociological view. According to the positivist empiricist view, it occurs through a rational, linear, and unilateral process of knowledge transfer from evidence *producers* to evidence *users*, a process often depicted as a pipeline starting with the generation of knowledge, through to its synthesis, and then its uptake or implementation (Rushmer et al., 2019). This understanding of KT traces its origins to the evidence-based medicine (EBM) and evidence-based practice (EBP) movements (Puljak, 2022) in which physicians and other healthcare professionals were called upon to be more proactive in searching, examining, critically evaluating scientific literature, and applying it to clinical practice. Emphasis was placed on empirical evidence from randomised control trials, observational studies, and meta-analyses and syntheses (Evidence-Based Medicine Working Group, 1992; Sackett et al., 1996). According to the sociological view, the uptake of evidence or knowledge into practice occurs through various channels among members of a social system and is mediated by the structure and norms of that system. Thus, the practice of scientific research (its institutions and norms) and its translation was understood in terms of the social and relational systems in which it is embedded, and hence KT was understood as a sociological phenomenon. Such a view traces its origins to Everett Rogers’ seminal “Diffusion of Innovations” theory first published in 1962 (Rogers, 2003).

Original conceptualisations of EBM and EBP acknowledged the importance of considering patients’ emotional and psychosocial needs and the effect of care relationships on care outcomes, but such considerations were not explicitly understood as “knowledge” relevant to KT. What thus emerged was a hierarchical view of knowledge that privileged the kinds of empirical evidence referred to previously, in which it assumed that non-application of such evidence constituted a failure of knowledge translation. It also entrenched scientists, medical experts, and healthcare professionals in a position of prescriptive authority over patients. Such a view eventually prompted criticisms of the EBM and EBP movements as being scientistic and contributing to the time taken for evidence to be translated into practice (Goldenberg, 2006; Walsh and Gillett, 2011; White and Willis, 2002). In contrast, the sociological view emphasised the importance of practicing effective scientific communication, which not only requires presenting the evidence but also understanding how recipients respond to the evidence and devising strategies accordingly to promote appropriate change. On this view, evidence is seldom transferred and adopted in a unilateral or prescriptive manner, but is presented, argued for, interpreted, understood, and adapted to, in a negotiated and cooperative matter. It also captures the idea that knowledge from the medical and empirical sciences are not the only forms of knowledge relevant to human beings and that their non-application does not constitute a lack of knowledge translation. Thus, the sociological view offers a broader conception of KT that captures the broader range of conditions under which various categories of knowledge are successfully and appropriately translated into practice and policy (Kitto et al., 2012).

The Canadian Institute of Health Research (CIHR), a federal agency responsible for funding health and medical research in Canada, developed a widely used definition of KT that has been recently refined in order to capture the broader system of complex interactions across multiple stakeholders (CIHR, 2020)^2^. As a result, it now aligns itself more closely with the sociological view and reflects an emerging view of KT in which it is understood as a complex, dynamic, and highly integrated sociological phenomenon, a view that is both descriptive and normative. A noteworthy example of this emerging view is the KT Complexity Network Model (KT-cnm) proposed by Kitson et al. (2018), which explicitly situates KT processes within broader systems and institutional contexts, emphasising the complex, dynamic, and often unpredictable interactions between multiple stakeholders across multiple sectors. It seeks to capture key aspects from the EBM and EBP view (i.e., rigorous standards of scientific practice and knowledge), and the sociological view (i.e., dialogue, negotiation, and pluralism). Other complexity-informed and integrative models of KT have also been recently proposed, such as the Context and Implementation of Complex Interventions (CICI) framework (Pfadenhauer et al., 2017), the Nonadoption, Abandonment, Scale-up, Spread, Sustainability (NASSS) framework (Greenhalgh et al., 2017), and the Successful Healthcare Improvement From Translating Evidence in complex systems (SHIFT-Evidence) framework (Reed et al., 2019). The emerging view of KT thus extends its scope and application beyond the relatively circumscribed healthcare domain into the broader social and public domain, reminding us of the challenges and complexities associated with the relationship between science and public policy.

In this paper, we begin by discussing a few examples of the emerging critique of the role that scientific evidence and expertise play public policy, and related critique of KT. We highlight the interplay of political, ethical, and epistemological arguments aimed at challenging the privileged status of science in policy and practice, and its epistemic primacy. We then turn our attention to the way that the emerging critique aligns with and informs the emerging view of KT. Here we use the KT-cnm as a case example due to the way it explicitly situates KT within broader sociocultural and public policy contexts, though we do not necessarily advocate for it, nor do we endorse it as the “correct” model. Following on from this we consider the implications of the emerging critique for our understanding and practice of KT. We point out that it raises a significant dilemma for KT, especially given the prevalence of post-truthism, populism, and identity politics in the current sociopolitical climate. We suggest that KT researchers should be cautious in how they respond to the emerging critique, and in particular, resist the temptation to adopt contentious epistemological views that may further fuel the problems of the current sociopolitical climate.

The emerging critique highlights genuine problems in how science has been and continues to be applied to matters of public concern and has much to offer by way of informing emerging KT perspectives. However, our point of departure is to question the extent to which it enables us to effectively address matters of public concern by drawing attention to the problems of the current sociopolitical climate, particularly its detrimental impact on public discourse, which has thus far received inadequate consideration. This is the focus of the second part of the paper where we discuss the nature of those problems and their historical antecedents in the hope that it can help us better understand the challenges that KT faces and inform potential solutions. Ultimately, our view is that KT must provide scope for epistemic standards to exist and accordingly for scientific evidence to occupy an appropriate position of epistemic primacy within public discourse. This necessitates developing and implementing strategies to promote conditions for a basic consensus on elementary matters of fact and value, which can lead to the understanding that scientific evidence carries significant weight when it comes to deciphering facts or truths about particular matters. Indeed, many aspects of the emerging critique and emerging KT perspective can inform such an approach, some examples of which are provided in the final section.

## Emerging perspectives

### The emerging critique

The emerging critique and KT perspective reflect concerns about how scientific evidence and expertise has been privileged at the expense of culturally situated knowledge and humanistic values. Critics view such privileging as an expression of scientism or positivism or as a way of promoting “the scientific logos” (Greenhalgh and Engebretsen, 2022; Ødemark and Engebretsen, 2022). Such labels denote the broad range of ongoing tensions, challenges, discontents, and disaffection associated with the role of scientific expertise and evidence in public policy, or what has been referred to as “the science-policy nexus” (Hoppe, 2005). These matters have been amplified recently due to concerns about the growing prominence and purview of the field of KT, and how its promise of accelerating the translation of evidence into practice will manifest in the public domain. The COVID-19 pandemic has served as a touchstone for such concerns, particularly given the associated scientific uncertainties, contestations, and controversy surrounding public policy decisions (Boschele, 2020; Caniglia et al., 2021; Greenhalgh and Engebretsen, 2022; Lohse and Bschir, 2020). It has led many, including KT researchers, to become more critical of the role of science in public policy and more explicit in advocating for a different attitude and approach to the science-policy nexus. The critique aligns broadly with the “critical theory” perspective in emphasising emancipation from dominance and oppression while also advocating for freedom and participatory democracy^3^. It consists of a range of arguments about the importance of adequate public engagement and consideration of the social, political, ethical, and humanistic dimensions of the science-policy nexus (Bohman, 2021; Cairney and Oliver, 2017, Carney and Bennett, 2014; Heinsch et al., 2023; Pedersen, 2014; Stengers, 2018; Engebretsen and Baker, 2022). They are often coupled with arguments drawing from the philosophy of science and sociology of knowledge, proffering a view of scientific evidence as provisional, contested, and existing among a plurality of views that some may regard as carrying equivalent epistemic weight.

It should be noted that some arguments are framed as “epistemological” in virtue of emphasising the importance and relevance of different kinds of knowledge and viewpoints, but are not arguments for their epistemic equivalence per se. For example, in their argument for “epistemic pluralism”, Lohse and Bschir (2020) highlight concerns about insufficient consideration of the socioeconomic and psychological impact of COVID-19 lock-down measures, and policy decisions based on uncertain epidemiological modelling and projections that reflect limited consideration of both scientific and non-scientific perspectives. The argument is essentially about ensuring that public policy measures adequately consider and address the relevant range of matters of public concern rather than taking “a myopic, epidemiology-centric description of reality that can lead to imbalanced policy decisions” (p. 3). The authors cite the work of Paul Feyerabend as their inspiration but do not adopt his more substantive and controversial doctrines of “epistemological anarchism” (the view that there is no and ought not to be any single scientific method that scientists adhere to), “theoretical pluralism” (the view that science is not fundamentally different from the arts and can only progress through embracing freedom of artistic expression and creativity), and ontological and epistemic relativism (Feyerabend, 1975; 1978; 1987). Other arguments consist of more substantive epistemological claims. For example, Caniglia et al. (2021), in their argument for a “new epistemology of science”, begin by pointing out that science is a “historically situated and constantly evolving process”, one that “has been and remains complicit in forms of historically entrenched systemic injustice” (p. 4). From this, they conclude that:

Equity, diversity, and inclusion should be recognised as fundamental values in science not only for ethical reasons, but also on epistemological grounds. There is in fact a deep connection between ethics and epistemology that needs to be rediscovered and put into practice. (Caniglia et al., 2021, p. 4–5)

Ethical arguments for greater inclusivity in scientific and policy discourse are relatively uncontroversial but to claim that the issue is also an epistemological is one is not and requires a substantive epistemological argument. Caniglia et al. (2021) use the example of the importance of qualitative methods in epidemiological enquiry to act as a counterbalance and complement to the dominance of quantitative methods, but in the absence of a justification for such a claim, it is merely an assertion of the epistemic equivalence between quantitative and qualitative evidence (or between objective and subjective accounts). Boschele’s (2020) argument for “epistemic democracy” has essentially the same substance and structure. It begins by challenging the assumed objective position of scientific expertise and the idea that scientific evidence can be viewed from the “apolitical lens of the Enlightenment values” (p. 2). Presumably, their assertion below describes not only *one* way of tracking truths and seeking the common good, but also the *preferred* way.

…the combination of knowledge provided by the modernist experts and the will of the people represented by democratic procedures is one way to track broadly and critically deliberated truths and seek common good. (Boschele, 2020, p. 2)

For such critics, the requirement that governments work closely with public health experts in dealing with the COVID-19 pandemic exemplifies the blurring of boundaries between science and politics, reiterating the view that science cannot circumscribe itself from the sociopolitical domain. This is taken by some to bear substantively on matters of epistemology, namely that there is an epistemic equivalence between scientific knowledge and various other viewpoints including ethical and political views. Those acquainted with the philosophy of science and sociology of knowledge literature will be familiar with this argument^4^. Various iterations of this argument underpin the emerging critique of KT, of which a notable example is Engebretsen et al.’s (2020) poststructuralist critique. Drawing from Derrida’s critique of textual interpretation, they claim that “knowledge has no transcendent status, but it is an immanent and integral part of a textual productivity” (p. 4). As a case example, they describe the experience of Trish Greenhalgh, a distinguished professor of primary healthcare sciences, who is also a vocal critic of the science-policy nexus. Professor Greenhalgh had undergone surgery to treat fractures to her arms and neck caused by a biking accident. She was advised by her doctor to avoid using non-steroidal anti-inflammatory drugs (NSAIDs) to treat pain based on evidence that such drugs caused delayed bone healing and bleeding during the post-operative period. Her doctor’s advice (characterised as “standard KT procedure”) was criticised for being based on equivocal evidence, for lacking transparency, and for ignoring patient particulars and contexts of injury, namely, that Professor Greenhalgh had previous experience of injuries and fractures which were treated with NSAIDs without adverse effects.

The case demonstrates that evidence cannot be detached from its various cultural and textual forms of production… Hence medical KT is a scientific and a cultural practice on equal terms… The lesson from this case is that evidence should be seen as a system, or a net of traces—in Latour’s idiom, it has a network character—converging in singular case histories (like Greenhalgh’s). (Engebretsen et al., 2020, p. 5)

The appeal to Latour’s Actor Network Theory (ANT) suggests an adoption of Latour’s relativist ontology and epistemology, implying that the referents of scientific statements and thus their epistemic status are constructed from the complex activities (semiotic activities) of networks made up of both human and non-human “actors” (Latour, 1996; 2012). More recently, Greenhalgh and Engebretsen (2022) have made “an urgent call for a pragmatist turn” in how we navigate the science-policy nexus and how KT ought to proceed. Their critique draws from a range of ideas associated with pragmatism, including the pragmatic theory of truth in which it is “found not in the quest for generalities and abstractions, but in the usefulness of a piece of knowledge for informing specific actions”, and a fallibilist view of science in which “its truths are plastic, provisional and open-ended” (p. 2). Such claims are, however, highly philosophically controversial, particularly given that they are associated with various forms of anti-realism and relativism for which there are familiar and established objections^5^. We do not wish to reiterate those objections here but highlight that what is at stake is no less than the view that a mind-independent reality exists about which facts or truths can be discovered that are not merely forms of epistemological expediency but reflect to some degree such a reality. It is important to understand how the emerging KT perspective reflects and embodies these views and the implications it may have for KT’s role in addressing matters of public concern. Thus, in the following section we provide a brief overview of the KT Complexity Network Model (KT-cnm), which serves as a case example of how the emerging critique has shaped the emerging view of KT.

### The KT-complexity network model (KT-cnm) and the emerging KT perspective

**Fig. 1:**
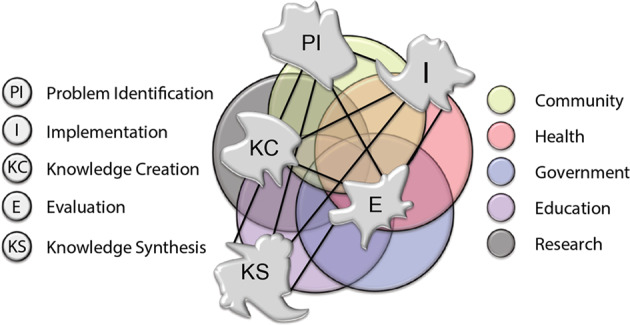
The KT Complexity Network Model (Kitson et al., 2018, p. 234). This figure is covered by the Creative Commons Attribution 4.0 International License. Reproduced with permission of IJHPM; copyright © IJHPM, all rights reserved. The IJHPM supports the Open Access initiative and the authors retain the copyright without restrictions. Abstracts and full texts (PDF format) of all articles published by the IJHPM are freely accessible to everyone immediately upon publication. Reusing and publishing IJHPM published articles (main text, tables, and figures) is permitted by following Creative Commons user license: https://creativecommons.org/licenses/by/4.0/. Users are free to copy and redistribute the IJHPM published articles in any medium or format under the Creative commons license terms and conditions, but need to provide the appropriate bibliographic citation of the IJHPM published articles in their works. https://www.ijhpm.com/journal/process?ethics.

The KT-cnm derives from an application of complexity science and network principles and expands on recent trends towards more integrated and dynamic conceptions of KT (Graham et al., 2006; Greenhalgh and Papoutsi, 2018; Kitson et al., 2013). So-called “evidence producers” (e.g., researchers, analysts, and associated institutions) and “evidence users” (e.g., practitioners, policymakers, and patients/consumers) are integrated into the model. It contrasts with models that treat them as distinct communities (the “two communities” view) in which the fundamental goal is to bridge the so-called “gap” between them (Rushmer et al., 2019). Such gaps are thus reconceptualised as discursive spaces or as “synapses of interaction and connectivity” in which the behaviour of multiple interacting stakeholders is shaped (Kitson et al., 2018). The KT-cnm also integrates five key KT processes captured within many existing KT models (Graham et al., 2006). Those processes are problem identification (PI), knowledge creation (KC), knowledge synthesis (KS), implementation (I), and evaluation (E). Previous KT models depict such processes as occurring linearly or cyclically, however, the KT-cnm views them as part of a dynamic, integrated, unpredictable, and complex process, supervening on the structural components of the entire system, which naturally behave and interact in ways that enable them to adapt and sustain themselves (Fig. 1). These structural components consist of individuals, groups, and networks (that interconnect with each other), whose decisions and actions are ultimately geared towards acceptance of, or adaptation to, various perturbations that arise from within or outside the system (e.g., the introduction of research evidence). Thus, drawing from complexity science, the overall system (including its components) is referred to as a complex adaptive system (CAS).

The accumulation of many simple and small-scale connections and interactions within a system can reach a critical threshold beyond which larger-scale complex group behaviours emerge (Braithwaite et al., 2020). Thus, for change to occur, the emphasis must be on leveraging those connections and interactions in ways that compel adaptive behaviour. Such an idea contrasts with the view that a determinative solution based on targeting particular system components to yield a predicted outcome exists. For this to happen the system must behave in predictable or rational ways, but this is not how complex systems operate. When there are multiple interconnected components, any single intervention directed at any particular component can lead to unpredictable outcomes elsewhere within the system. This also contrasts with the view that behaviour change can occur through centralised control, which is often not the case and can be counterproductive within complex systems, especially when the system components view the control mechanisms (e.g., rules, laws, governance) as maladaptive. For example, it can lead to the emergence of resistance and counter-movements, which find ways to adapt in accordance with their own interests and agendas. As Braithwaite (2018, p. 2) points out, “change is accepted when people are involved in the decisions and activities that affect them, but they resist when change is imposed by others”. This raises an important consideration. Individuals, groups, and networks have their own goals and interests and tend to operate within their own “silos” where there is more likely to be a shared understanding of the problems they face and the approach to addressing them. When the system involves a broader range of stakeholders there are more likely to be differences in goals, interests, understandings, and approaches, which can cause tension and conflict, including conflict with the originally formulated goal of KT itself.

Thus, the key to achieving KT outcomes is to promote interconnectivity and synergy among stakeholders so that a shared or mutually acceptable understanding of the issues emerges. This entails promoting a basic level of consensus on the nature of the problem, the relevant evidence, the benefits of accepting and adopting that evidence, and the approach needed to move the evidence into practice. Where such a consensus is impossible, developing an approach that appeals to the different ways stakeholders understand the problem is crucial. Promoting this type of interconnectivity and synergy among stakeholders is a highly demanding task, requiring continuous facilitation, monitoring and evaluation. Opportunities for early engagement and commitment between various stakeholders (across multiple sectors) must be seized upon so that stakeholders do not adapt to different perturbations in a siloed manner but instead co-adapt to those perturbations and thus co-evolve in connected and synergistic ways. Such co-adaptation and co-evolution will then encompass how they understand the nature of the problem and the evidence that bears on it. Where there had been conflicting views and tensions, the co-adaptive process entails a shared understanding of the issues or a mutual acceptance of multiple understandings.

Coordination and effort are required to maintain, strengthen, and expand such interconnectivity and synergy, especially through sustained communication and dialogue that includes those beyond one’s immediate community. Barriers that prevent or limit such communication and dialogue (e.g., geographical location, lack of shared understanding, differences in vocabulary, and competing agendas) must therefore be identified and carefully navigated. Often, within stakeholder groups there are individuals with certain characteristics, attributes and skills who interact more extensively within their own and between various other groups. In doing so, they perform the crucial role of promoting interconnectivity and synergy by facilitating engagement and dialogue and overcoming barriers. KT models conceptualise such individuals as “knowledge brokers”, “facilitators”, or “boundary spanners” (Cranley et al., 2017; Elledge et al., 2018; Harvey and Kitson, 2016; Long et al., 2013; Rushmer et al., 2019). Using the language of complexity science and network analysis, Kitson et al. (2018) refer to them as “hubs”, denoting their well-connected position in the network. For our purposes, however, we refer to them simply as “champions”, whose role is essentially to drive the KT process by helping stakeholders to co-adapt to the unpredictability of the complex systems they are in.

In summary, the KT-cnm seeks to offer a more sophisticated understanding of how and why evidence moves within and through complex systems to where it can be put into practice. It embodies much of the emerging critique discussed previously, particularly the problems and challenges arising from inadequate consideration of the cultural and humanistic dimensions of KT and the need for a more integrated, engaged, and inclusive approach. It purports to neither assume nor create knowledge hierarchies and neither prescribe nor privilege particular kinds of knowledge. It acknowledges that hierarchies of knowledge, power imbalances among stakeholders, coercive control, and unilateral approaches are problematic and counterproductive. It captures the idea that a shared or mutually acceptable approach to addressing complex problems can only be achieved through a cooperative process involving dialogue and negotiation. It advocates for scientific research activity, ideas, and proposals to be discussed, tested, and negotiated within context involving multiple stakeholders from multiple sectors at multiple times. Thus, it adopts a pragmatic stance whereby knowledge is understood in terms of its creation, purpose, and efficacy within relevant contexts. In this regard it also constitutes a normative framework for promoting appropriate change in settings where multiple stakeholders operate under complex personal, social, cultural, and institutional constraints. Its central theme is co-evolution, something that is achieved when there is interconnectivity and synergy between all relevant stakeholders. To achieve such a state there must be: (1) early engagement of stakeholders across multiple sectors; (2) a shared understanding of the relevant issues or mutual acceptance of pluralities; (3) champions to promote engagement, interactivity, shared understandings, co-existence of differing viewpoints, and movement of knowledge within and across multiple stakeholder groups; and (4) continuous monitoring, evaluation, and maintenance of the quality of engagement and interactivity between stakeholders.

## A dilemma for KT

### The epistemic primacy of science

KT as a discipline is primarily concerned with the application of scientific evidence in practice and KT researchers themselves are typically scientists whose views on relevant matters are shaped by the available scientific evidence, associated methods and overall disciplinary training. They may recognise scientific evidence as provisional, contested, and existing alongside many other views that should be afforded equal consideration, but such recognition does not entail that they regard all viewpoints as having the same epistemic status. Indeed, in KT there is an implicit assumption that scientific methods and evidence occupy a position of epistemic primacy, which can be understood in terms of two related ideas, namely, scientific realism and the normativity of science^6^. The former refers to the view that a mind-independent reality exists and that through science we can (at least some of the time) arrive at true descriptions of it. The latter refers to how arriving at such truths depends on standards of practice and methods that are conducive to producing true descriptions of reality. In this regard, scientific hypotheses and theories that have been rigorously formulated, tested, and analysed, using appropriate methods, have the kind of epistemic status that theories which have not undergone such rigorous testing and analyses (or have failed them) lack. This is not to suggest that scientists assume the truth of their hypotheses and theories, that their methods are infallible, or that scientific knowledge ought to supplant all other relevant facts or considerations. Rather, it highlights how science is predicated on the view that there are objective facts to be discerned about reality and that certain methods are better at arriving at such facts than others. Thus, how KT is understood and practiced may be undergoing an evolution towards more complex, sociologically informed, and inclusive processes, but it is still underpinned by the assumption of the epistemic primacy of science and the epistemic status of scientific evidence.

Insofar as the emerging critique uses the language of “pragmatism” and “pluralism” in what might be described as a folk or colloquial sense (perhaps as a tactical manoeuvre to facilitate KT outcomes), then it remains compatible with such a view of KT. Critics, however, may view it as a cynical and surreptitious manoeuvre aimed at upholding and promoting the privileged status of science. Insofar as “pragmatism” and “pluralism” is used to denote a more substantive epistemic position associated with the philosophy of science and sociology of knowledge perspective, then it is no longer compatible with such a view of KT and faces a range of philosophical objections (Boghossian, 2006; Nagel, 1997; Putnam, 1982a; Seidel, 2014) of which a common objection is that of self-contradiction.

…its thesis of the context-dependency of all knowledge must also apply to the sociology of knowledge itself, the sociology of knowledge makes a general truth claim which it at once denies. (Meja and Stehr, 1988, p. 264)

From a practical standpoint, what is perhaps more concerning is that regardless of the kind of “pragmatism” or “pluralism” adopted, KT may have limited utility in addressing matters of a national and global scale that require immediate action, such as the COVID-19 pandemic and anthropogenic climate change. In the current sociopolitical climate, people have become increasingly discontent and disaffected by evidence-based policy responses while increasingly amenable to a variety of competing and often controversial perspectives. For them, the epistemic status of scientific evidence is becoming increasingly irrelevant. A fundamental part of the problem is that scientific evidence may be highly nuanced, provisional, and in some cases highly contested, and thus a shared public understanding of the basic facts pertaining a particular matter may not exist to begin with. This can cause people to deny that a particular matter exists or requires addressing at all (e.g., whether climate change is real and caused by human activity, whether social distancing, mask-wearing and vaccination protects against COVID-19 and reduces its transmission). How people understand and receive scientific evidence is not only influenced by its perceived economic impact, but also by how it bears on their moral values and cultural norms. Difficulties arise when scientific experts and policymakers act in ways that challenge or undermine people’s values and belief systems, and particularly if it is done in haste and without adequate public consultation or dialogue. It can also lead to backlash and distrust in the very institutional systems responsible for addressing matters of public concern (Smith, 2013).

The emerging critique rightfully highlights the relevance and importance of these considerations and the problems that KT faces if it neglects such considerations. However, it fails to acknowledge the difficulty and dilemma of having to accommodate such considerations in the current sociopolitical climate while also bringing the relevant science to bear on matters of public concern, particularly highly complex national and global scale “wicked problems” such as the COVID-19 pandemic and anthropogenic climate change (Rittel and Webber, 1973). In these contexts, adopting a pragmatist or pluralist stance is unlikely to succeed because there is far too much pluralism to be navigated and reconciled for a pragmatic solution to be developed and implemented on time. All things considered, it is difficult to fathom how scientific evidence could not play a central role in informing how we address such wicked problems, for example, applying standardised public health measures to reduce COVID-19 spread (Ayouni et al., 2021; Talic et al., 2021) or adopting the recommendations of the Intergovernmental Panel on Climate Change (IPCC) (IPCC, 2022). For KT to relinquish such a role for scientific evidence or reduce it to the status of one perspective among many of equally validity is for KT to be indifferent to the urgency and sociopolitical context of such wicked problems.

### The current sociopolitical climate

Consultation, engagement, and dialogue with all stakeholders, and co-adaptive responses are essential features of the emerging KT perspective. However, such activities cannot proceed without a basic level of prior consensus on certain fundamental facts and values, a consensus that is thus axiomatic to the KT process. For many of the wicked problems we face, no such consensus exists. Aside from the examples of the COVID-19 pandemic and anthropogenic climate change, this situation characterises many ongoing social policy matters regarding healthcare, poverty, immigration, racial and gender equality, LGBTQ rights, and of particular note, abortion rights and gun-control in the US. Policy responses aimed at addressing those issues are highly controversial often resulting in public backlash and protracted battles between those in favour of and against the responses. Recently, the issue of abortion rights and gun-control in the US has demonstrated how powerful lobby groups, private corporations and political opportunists are able to influence legislative and judicial outcomes in ways that contravene the democratic will of the public (Molla, 2022; Ziegler, 2022). Those in favour of abortion rights and gun-control may argue that in addition to democratic values, scientific evidence concerning health risks of giving birth, consequences of raising children in poor socioeconomic conditions, and associations between access to guns and prevalence of gun-related homicides and mass shootings, ought to bear on the policy response. However, for many who are opposed to abortion rights and gun-control, these issues are viewed through an ideological and moral lens. The battle lines on these matters had been drawn long ago as part of the “culture wars” (Hunter, 1991) and now similar battle lines and political and cultural divisions have emerged with regard to the COVID-19 pandemic and anthropogenic climate change (Dunlap, 2013; McLamore et al., 2022; Rubin, 2016; Thagard, 2021). This is particularly concerning given the scope and urgency of those problems and need to have consensus on relevant basic facts and values in order to appropriately address them.

The cornerstone of liberal democracy is a healthy functioning public sphere in which public consultation, engagement, discussion, and debate on matters of public concern can take place. It is through such a means that we may achieve a basic level of democratic consensus necessary to begin addressing such wicked problems. However, recent historical conditions, which we will discuss in the following sections, have adversely affected the public sphere, making it increasingly difficult to promote democratic outcomes and the public good through public discourse. Powerful actors are now able to challenge basic facts and scientific evidence, sow distrust and discord among the public, and leverage existing and emerging political and cultural divisions to influence public policy in accordance with their own interests. This has the effect of undermining the ability of the public to be informed on relevant matters, to engage in appropriate public discourse, and to express their democratic will. A key element of this sociopolitical situation is captured by Oxford Dictionary’s 2016 word of the year, ‘post-truth’, which it defines as “relating to or denoting circumstances in which objective facts are less influential in shaping public opinion than appeals to emotion and personal belief” (Coughlan, 2017).

Thus, the dilemma for KT researchers (and the many who place trust in scientific evidence) is that on the one hand the emerging perspective opposes a unilateral approach that simply imposes outcomes on the public. Such an approach is likely to be counterproductive and may also encourage the public to become increasingly sceptical of scientific expertise and governments while increasingly susceptible to post-truth narratives. On the other hand, given the urgency of wicked problems like COVID-19 and anthropogenic climate change, and the difficulty of navigating the complex interplay of politics, economics, vested interests, and sociocultural divisions, the tactical manoeuvre of engaging with and integrating the broad range of public viewpoints on these wicked problems is also unlikely to succeed and may be counterproductive. Such an approach requires a level of prior consensus on certain basic facts and values pertaining to those wicked problems, for example, facts that entail recognition of the problem’s existence and its most elementary nature, and values such as knowledge, truth, democracy, the public good, and survival of species. In light of these considerations, we argue that the emerging KT perspective should be geared towards promoting the conditions for such a basic consensus to emerge, and more generally, redressing the problems of the current sociopolitical climate rather than accommodating them. This would enable future KT approaches to be more effective in addressing such wicked problems. Consideration of the current state of the public sphere and public discourse, and its historical antecedents can help to inform the approach.

## KT in a post-truth political economy

### A post-truth public sphere

The ideal of a healthy and functioning public sphere is captured by Jurgen Habermas’s notion of the “bourgeois public sphere”, which he described as “a sphere of private people come together as a public” (Habermas, 1989, p. 27). The emergence of the bourgeois public sphere during the late modern period was made possible by the social and communicative infrastructure of the time, namely, coffee houses and various other physical places (“salons”) for gatherings and voluntary associations, as well as print media and the public press, which enabled ideas and information to be disseminated to the wider public. This infrastructure facilitated informed discussions and debate on a range of topics, particularly literature, the arts, and politics. The notion of the public sphere thus denoted a space where all could participate as equals regardless of occupation, class, or caste, enabling collective self-determination and democratic freedom. It also enabled people to develop the skills necessary for fruitful engagement and thus gave rise to new norms of discourse and debate. Habermas also emphasises the role of “communicative rationality”, which he describes as a form of reason and discourse that promotes shared understandings and consensus, contrasting it with “strategic rationality”, which is aimed at promoting personal interests and agendas. Habermas believed that communicative rationality was possible because people have an inherent ability to evaluate each other’s viewpoints using appropriate discursive and dialectical standards that involve reason and evidence, and an inherent ability to socialise and to thus reach common ground on social and political matters (Habermas, 1984; 1996).

However, with the rise of industrial capitalism throughout the 19^th^ and early 20^th^ centuries, the public sphere was transformed in ways that departed from the bourgeois public sphere and its associated liberal democratic ideals. Social power was increasingly concentrated in the private hands of large corporations through market means and public policy (i.e., privatisation). The economically marginalised, expecting universal accessibility, equal opportunity, and entitlement to political participation, would respond primarily through political means. For them, the solution to disparity in wealth and social power was a stronger state. However, those with wealth and social power would also respond by leveraging their relationship with the state to gain political power, which Habermas (1989) characterises as the “refeudalization” of civil society, stating that it had “gradually destroyed the basis of the bourgeois public sphere” (p. 142). By the middle of the 20^th^ century the public sphere had been further transformed by wealthy corporations that had major social, economic, and political influence, especially through mass media and mass marketing. The resultant expansion of consumer culture also meant that citizens would be less concerned with discussing public matters and more concerned with pursuing private interests. Thus, on the one hand, people began to abstain from literary and political debate, while on the other hand, mass media, popular culture, and various corporate entities, gradually took over this role, framing and shaping public discourse, public opinion, and ultimately public policy. Indeed, public discourse would itself become a commodity for the consumer market, taking on a private leisurely quality, losing its moral and public substance.

What can be posed as a problem is defined as a question of etiquette; conflicts, once fought out in public polemics are demoted to the level of personal incompatibilities. Critical debate arranged in this manner certainly fulfills important social-psychological functions, especially that of a tranquilising substitute for action; however, it increasingly loses its publicist function. (Habermas, 1989, p. 164)

The emergence of the “culture wars” in the mid to late 20^th^ century, and associated identity politics between so-called “liberals” and “conservatives” (particularly in the US), was a consequence of the transformation of the public sphere described by Habermas. It was extremely difficult for liberals and conservatives to resolve issues that defined their war (e.g., abortion, sexual identity, marriage, family, race, immigration, gun control, and popular culture). The public sphere became a sphere of parochialism in which partisans formed political, corporate, and legal allegiances to have their views prevail using increasingly strategic and rhetorical forms of public discourse. Nowadays, liberals and conservatives alike continue to align themselves with institutions and political parties that embody and seek to normalise their values, however, the divisions seem to have become further entrenched and bound by ideology. For example, new progressive social movements have emerged (e.g., Black Lives Matter, Me Too, LGBTQ rights, racial equality, and the Climate Movement) that express themselves through protestation and censorship or what is referred to as “cancel culture”. Conservatives respond by appealing to various notions of freedom of speech, freedom of religious expression, civil liberties, and freedom from government oppression, indicating more explicit incorporation of libertarian, nationalist, anti-globalisation, and anti-regulation perspectives into their ideological purview. Adding to the divisions are the problems of income inequality and their negative impact on the working and middle class. This was particularly felt during the 2008 global financial crisis and has led to increasing resentment and disaffection with government and an increasingly critical stance towards globalisation, immigration, and multiculturalism. Populist figures who openly challenged liberal institutions and movements while simultaneously repudiating the perceived weaknesses of the conservative establishment were thus able to win the approval of a large proportion of the working and middle class, the most noteworthy example being Donald Trump who became the 45th US president. Fox News had already established itself as the preferred and authoritative source of news and information for conservatives, but populist media figures and public intellectuals promoting ideas that challenged existing and emerging liberal movements also began to emerge. As Beinart (2016) points out, “Trump offers intellectuals the chance to speak for the energised masses and thus to make themselves relevant beyond their salons”.

The nature of the public sphere and public discourse continues to change at a rapid pace due to the digital and information revolution. Various new platforms, movements, and institutions (e.g., Twitter, Facebook, YouTube, Google, and various online news outlets and think tanks) that are underpinned by market driven approaches have further enabled the proliferation of social movements devoted primarily to influencing and shaping rather than discussing and debating public policy (Stewart and Hartmann, 2020). Given the current tendency towards partisanship, homophily, and identity politics, and bolstered by internet algorithms that further “personalise” our experience, this new public sphere becomes increasingly fragmented, segregated, and rife with echo chambers, while also creating new social inequities associated with digital access and digital literacy. Unfettered by traditional norms of public discourse, and mostly unregulated, it is now highly conducive to private people, organisational entities, and increasingly non-human entities (e.g., internet algorithms, “bots”, AI and machine learning) operating via strategic rationality, leveraging their outreach and influence to further their own interests. The methods used are increasingly deceitful, often involving the spread pseudo-scientific, anti-scientific, and conspiratorial views. Many are aimed at promoting conservative viewpoints, which is why, for example, we see a relationship between conservative distrust in scientific expertise and opposition to COVID-19 preventative measures in the form of anti-vaccine sentiment and accusations of oppression and authoritarianism levelled at governments (McLamore et al., 2022; Thagard, 2021). Similarly, we see conservative opposition to policy aimed at reducing carbon emissions and conversion to renewable energy sources linked to the denial of the anthropogenic contribution to climate change (Dunlap, 2013; Rubin, 2016), much of which recapitulates the tobacco industry’s approach in dealing with increasing public awareness of the harmful effects of cigarettes during the mid-90s (Brandt, 2012). Given that it was relatively easy for Trump and his media allies to convince many of his supporters that the 2020 US Presidential election was “stolen”, it is no surprise that institutions (including government) whose function is to promote the public good would also be viewed with suspicion.

The emerging critique and emerging KT perspective provides insight into how such a sociopolitical situation can arise. When public institutions impose coercive measures that have significant personal impact without genuine or adequate public consultation or negotiation, seeds of distrust are sown, and arguments or policy based on scientific evidence lose their epistemic and moral weight. Complex systems (e.g., liberal/conservative movements and institutions) will adapt according to their own goals and interests, and in competition with other systems, using increasingly strategic and unilateral means if necessary. It becomes easier to make the case that a particular policy response is merely a form of coercive control imposed for economic or political gain rather than for the public good, especially if there is perceived or actual negative impact on particular individuals. Ultimately, when a disaffected public no longer trust that institutions like universities, governments, and regulatory bodies exist to promote the public good, but instead exist to promote their own private interests, the epistemic status of scientific evidence becomes moot and post-truthism flourishes.

### What does this mean for KT?

The contemporary public sphere now embodies various social, cultural, political, economic, and historical conditions that make it increasingly difficult for relevant scientific evidence to appropriately bear on public policy. This is highlighted by the examples of the COVID-19 pandemic and anthropogenic climate change where the necessary preconditions (i.e., a basic consensus on relevant facts and values) for emerging KT approaches to resolve such wicked problems do not exist. Champions to facilitate engagement, interactivity, and to mobilise relevant knowledge across multiple stakeholder groups in the public sphere are lacking. Traditionally, such roles would have been fulfilled by the media, public intellectuals, captains of industry, and elected public officials, but many have become or have been displaced by partisans and populists. Public broadcasting and public interest journalism has lost much of its outreach and authority, especially after being tainted with the label of “fake news” by conservative media and conservative populists. Dealing in facts and engaging stakeholders may facilitate consensus and arbitrate over conflicts, but this can be difficult if facts are highly complex and inadequately communicated. It is near impossible if facts no longer matter or are considered to be on equal epistemic footing with contrary viewpoints, especially those associated with post-truth narratives.

Scientific evidence plays an indispensable role in addressing many of the wicked problems we face, particularly with regard to how we understand the nature of the problems, the threat they pose, and potential solutions. However, in order for scientific evidence to play such a role, it must first be distinguishable in some way from the myriad viewpoints that exist, especially those that are simply untrue and perhaps dangerous. Thus, we point out that scientific evidence naturally distinguishes itself in virtue of its epistemic status, which as discussed in a previous section, is simply the view that the methods of science are the best methods we have for discovering certain truths or facts about the world. It does not entail that scientific evidence is infallible or that it ought to supplant all other relevant facts or considerations. Indeed, the emerging critique makes a compelling case for the need to consider facts about culture, tradition, and values alongside relevant scientific evidence, but as we have argued in this paper, the application of such a perspective in the current sociopolitical climate makes it increasingly difficult for scientific evidence to bear on matters of public concern. Therefore, KT must ensure that there is scope for scientific evidence to occupy an appropriate position of epistemic primacy within public discourse.

Indeed, many of the basic principles of the KT-cnm can be applied towards achieving such outcomes and also align with recent proposals aimed at addressing the problems of the current sociopolitical climate. For example, reflecting the importance of early engagement with stakeholders, many have advocated for an educational response, in the form of embedding scientific literacy, critical thinking skills, and civic education programs in primary and secondary school curricula (Barzilai and Chinn, 2020; Cohen et al., 2021; Feinstein and Waddington, 2020). Some also highlight the need for innovation in education to find more effective ways of promoting those skills (Chinn et al., 2021; Valladares, 2022) and promoting greater political participation (Bauml et al., 2021; Weinberg, 2022). This can help facilitate the potential of “citizen science” approaches to generate policy-relevant recommendations, which have been shown to successfully challenge and change entrenched views on issues such as vaccination (Parrella et al., 2016; Wise, 2021). An adequately educated and informed population can also motivate appropriate action and community leadership on relevant issues. For example, while nations struggle to reduce their carbon emissions, there has been a groundswell of activity at the level of local jurisdictions (states and cities), organisations, companies, and individuals, who have declared their commitment to reach goals of net-zero (Nguyen, 2020). There is also increasing awareness of what can be done at the level of consumer behaviour (e.g., diet and travel) (Marteau et al., 2021). Such activity, engagement, and exchange of ideas at the individual and local community level can empower people to become champions in their own right and enable them to have greater influence at the structural and policy level, which is where real and impactful change happens^7^.

The role of champions also extends to monitoring, evaluating, and maintaining quality of engagement and interactivity. As many have pointed out, there is a need for a healthy media landscape (including social media) to facilitate appropriate public discourse and debate, and to act as a counterbalance to the prevalence of misinformation (Dobbie, 2022; Harrison, 2019; Sweet et al., 2020). These issues are particularly relevant in countries where media corporations have major social and political influence (e.g., the Murdoch press, Facebook, and Twitter). Public intellectuals, through their expertise, personality, and ability to attract a following, have an important role to play in this too, especially in educating the public, responding to misinformation and disinformation, and acting as a counterbalance to populist figures who in the absence of competition have enjoyed great returns in the marketplace of ideas and “alternative facts”. Some have called for more academics to venture beyond their ivory towers and take up this challenge (Alderman and Inwood, 2019; Ford and Jandric, 2019; Giroux, 2012). At a time when the views of scientific experts are regularly and publicly challenged, there is a growing need for them to explain and defend their views in the public sphere, which inevitably commits them to public discourse (Gattone, 2012; Lavazza and Farina, 2020). It is also increasingly difficult for them to assume the position of neutral observers while they are continuously undermined by populist figures and conservative media, and while the capacity for their institutions to promote the public good are diminished by neoliberal inspired corporatisation (Baltodano, 2012; Broom, 2011; Fatsis, 2018; Mintz, 2021). Ultimately, without a greater presence of qualified intellectuals in the public sphere who are willing to go toe-to-toe with the populists, we end up with what Robinson (2018) describes as the intellectuals we deserve.

## Conclusion

The recent evolution in our understanding of KT demonstrates a recognition of the complex sociological nature of the problems that KT aims to address. Emerging KT perspectives such as the KT-cnm help us understand how complex systems are constituted, how they behave, and how promoting interconnectivity and engagement within and between systems increases the likelihood of co-adaptive and co-evolutionary outcomes. The emerging KT perspective thus provides a framework for matters of public concern to be addressed in a collaborative and inclusive way. However, with regard to wicked problems that require a rapid collective response, it may be ineffective and potentially counterproductive, especially in the current sociopolitical climate. For posterity, we argue that KT must advocate for epistemic standards, and accordingly, for scientific evidence to occupy an appropriate position of epistemic primacy within public discourse. This can be achieved by tailoring emerging KT approaches towards redressing the problems of the current sociopolitical climate rather than accommodating them. Only then can a consensus on relevant elementary facts and values emerge, and the preconditions for effective KT established. The intention here is neither to affirm nor promote the privileged status of science, nor to surreptitiously impose scientific evidence or scientific standards of practice on the public. Rather, it is a necessary response to a post-truth political economy, one in which democratic outcomes and the public good are too easily undermined by extant and emerging power structures that are now able to leverage the highly problematic idea that the epistemic status of scientific evidence is irrelevant or unfounded.

## Data Availability

No data were generated or analysed.

## Footnotes

1 Throughout the paper we use the terms “knowledge” and “evidence”interchangeably but recognise that there are conceptual differences based on the tripartite model of knowledge as true justified belief.

2 The CIHR’s current working definition describes KT as a “dynamic anditerative process” which “takes place within a complex system of interactions between researchers and knowledge users” (CIHR, 2020).

3 These concerns have set the agenda for current and future scholarship on this topic. We recommend those interested familiarise themselves with the special collection on“Challenging medical knowledge translation” in *Humanities and Social Sciences Communications* (https://www.nature.com/collections/eddejjibjd), and the special issue on the “Sociological Aspects of Knowledge Translation” in *Health Sociology Review* (https://www.tandfonline.com/toc/rhsr20/32/1).

4 The argument is that science is a cultural and institutional practice, underpinned by social and historical contingencies and so the epistemic status of scientific knowledge must also be underpinned by such contingencies. Therefore, science cannot claim to have an epistemic status independent of such contingencies (Berger & Luckman, 1966; Bloor, 1976; Collins,^1985^; Latour & Woolgar, 1979; Merton, 1973).

5 For a comprehensive and accessible overview of objections to the range of metaphysical and epistemological claims associated with the emerging critique, see Blackburn (2005) and Boghossian (2006).

6 Our goal is not to argue for scientific realism or to justify the epistemic primacy of science but to clarify those notions so as to inform the emerging critique. For the seminal articulation and defence of such positions, see Boyd (1983) and Putnam (1978; 1982). For a more recent and accessible view, see Blackburn (2005).

7 Individual level contributions to carbon emissions pale in comparison to those of the fossil fuel industry, and it should be noted that British Petroleum (BP) promoted and popularised the idea of an individual “carbon footprint” in a surreptitious effort to shift responsibility (and blame) onto consumers (Solnit, 2021).
